# Supplementary material for: Auditory input enhances somatosensory encoding and tactile goal-directed behavior
Source: Nat Commun. 2021 Jul 23;12:4509. doi: 10.1038/s41467-021-24754-w (PMC8302566; doi:10.1038/s41467-021-24754-w)
Supplement: Supplementary file 1 — Supplementary Information [file 41467_2021_24754_MOESM1_ESM.pdf]

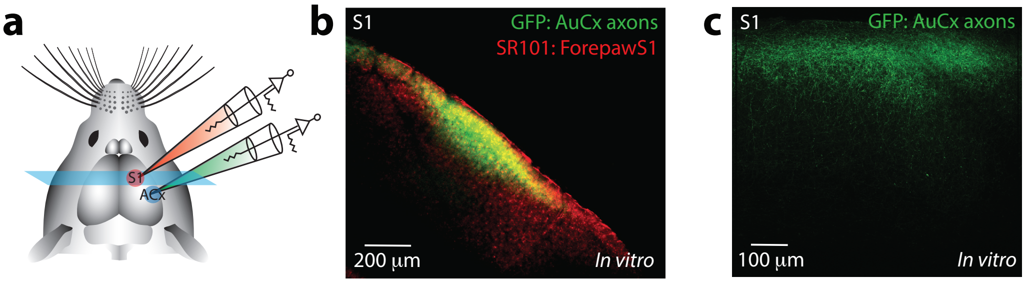

Supplementary Figure 1. The auditory cortex sends direct axonal projections to the primary somatosensory cortex. a. Schematic of experimental paradigm. GFP-tagged AAV was injected into the auditory cortex and the glia cell marker SR-101 was injected into forepaw S1. **b.** Example brain slice of the somatosensory cortex from a mouse with dual injections as in (a). Green, axonal projections from the auditory cortex. Red, glia cells. **c.** Example illustrating that axonal projections from the auditory cortex (green) targeted the upper layers of the primary somatosensory cortex.

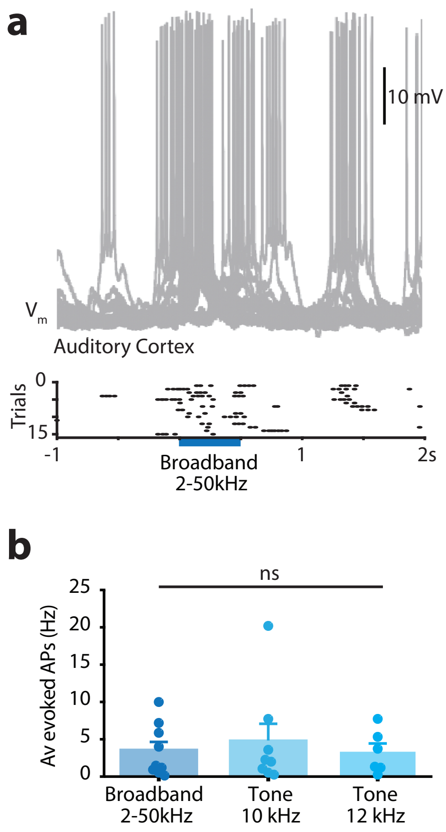

Supplementary Figure 2. Neurons within the auditory cortex robustly respond to broadband auditory stimulus. a, Patch clamp electrophysiological recordings were performed from layer 2/3 pyramidal neurons in the auditory cortex of urethane anaesthetized mice. Broadband auditory stimuli (2 - 50 kHz, 500 ms) reliably evoked action potentials. Top, Overlay of somatic voltage traces during auditory stimuli. Bottom, Raster of action potentials. **b,** The average evoked action potentials (APs) did not significantly differ during the presentation of broadband auditory stimuli (2 - 50 kHz; 3.48 ± 1.17 APs; n = 9 neurons, 3 mice), 10 kHz pure tone (500 ms; 4.71 ± 2.37 APs; n = 8 neurons, 3 mice) and 12 kHz (500 ms; 3.27 ± 1.18 APs; n = 6 neurons, 3 mice; p = 0.98). Kruskal-Wallis test. Error bars represent S.E.M.

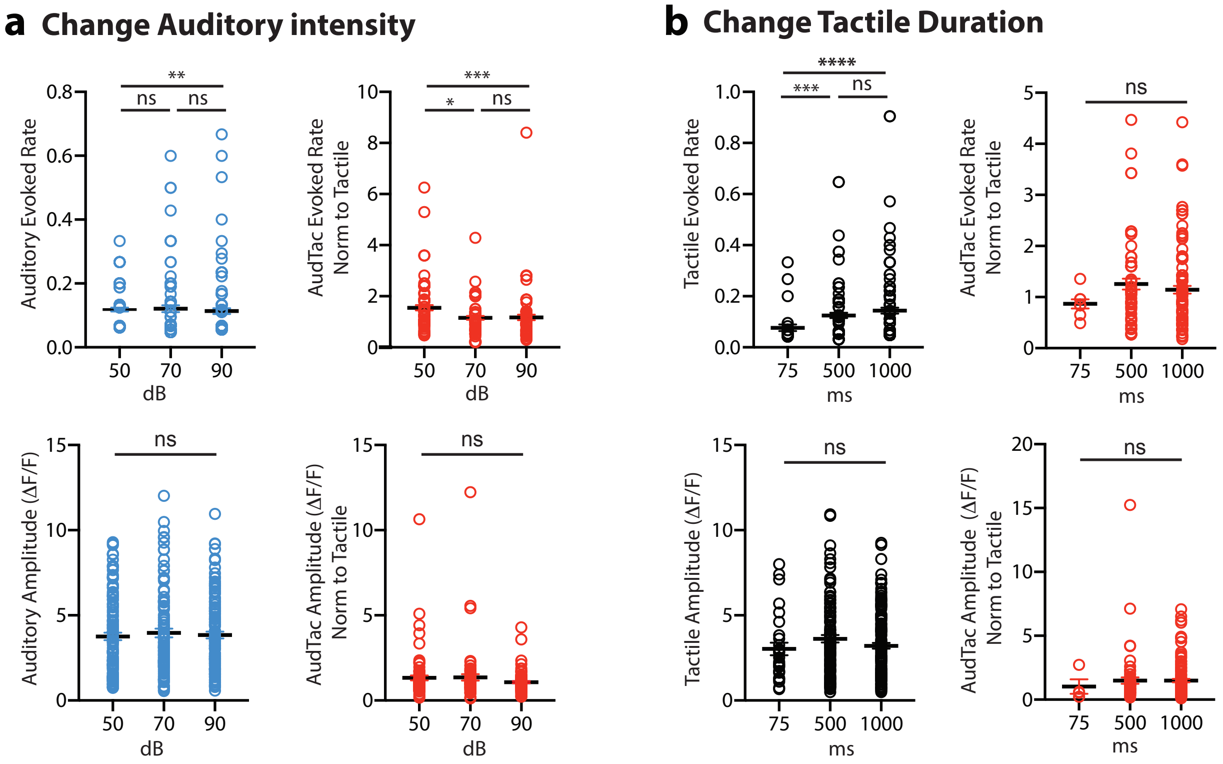

Supplementary Figure 3. The influence of changing auditory intensity and tactile duration on L2/3 pyramidal neuron dendrites. In vivo two-photon Ca2+ imaging was performed in the tuft dendrites of L2/3 pyramidal neurons in naive mice previously injected with the genetic Ca2+ indicator, GCaMP6f. **a,** The amplitude and rate of evoked dendritic Ca^{2+} transients was recorded in response to broadband auditory stimuli (2 - 50 kHz; 500 ms) of varying intensities (50, 70, 90 dB) alone (blue) and paired with tactile stimuli (red; 200 Hz; 500 ms). Tactile, n = 121/99/125 dendrites; AudTac, n = 84/69/87 dendrites; 4 mice. * p = 0.03; ** p = 0.004; *** p =0.0001. **b,** The amplitude and rate of evoked dendritic Ca2+ transients was recorded in response to tactile stimuli (200 Hz) of varying duration (75, 500, 1000 ms) alone (black) and paired with broadband auditory stimuli (red; 2 - 50 kHz; 500 ms; 70 dB). Tactile, n = 29/107/139 dendrites; AudTac, n = 8/66/113 dendrites; 4 mice. *** p = 0.007; **** p < 0.0001; ns: p > 0.999 . All statistical analysis was performed using a one-way Kruskal-Wallis test followed by a Dunn's multiple comparisons test.

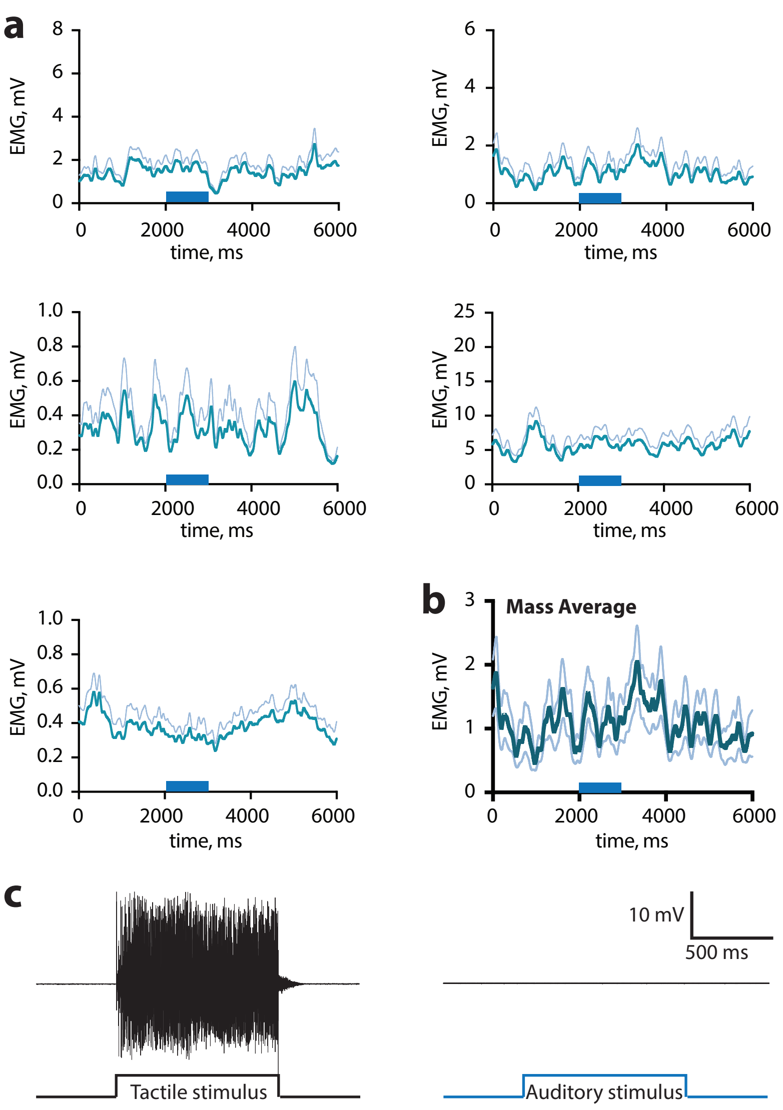

Supplementary Figure 4. Auditory stimulus does not cause body movement (startle response) nor movement of the forepaw stimulator. a. Average examples of nuchal EMG recordings from two electrodes bilaterally inserted in the neck muscles of 5 mice. Auditory stimulus presentation, blue line. **b.** Average EMG recordings during broadband auditory stimuli (2 - 50 kHz, 60 dB; n = 5 mice). Auditory stimulus presentation, blue line. **c.** Vibration of the forepaw stimulator was measured by creating an electrical circuit with the stimulator during (left) tactile stimulus (200 Hz; 1 s) and (right) auditory stimulus (2 - 50 kHz, 60 dB; 1 s).

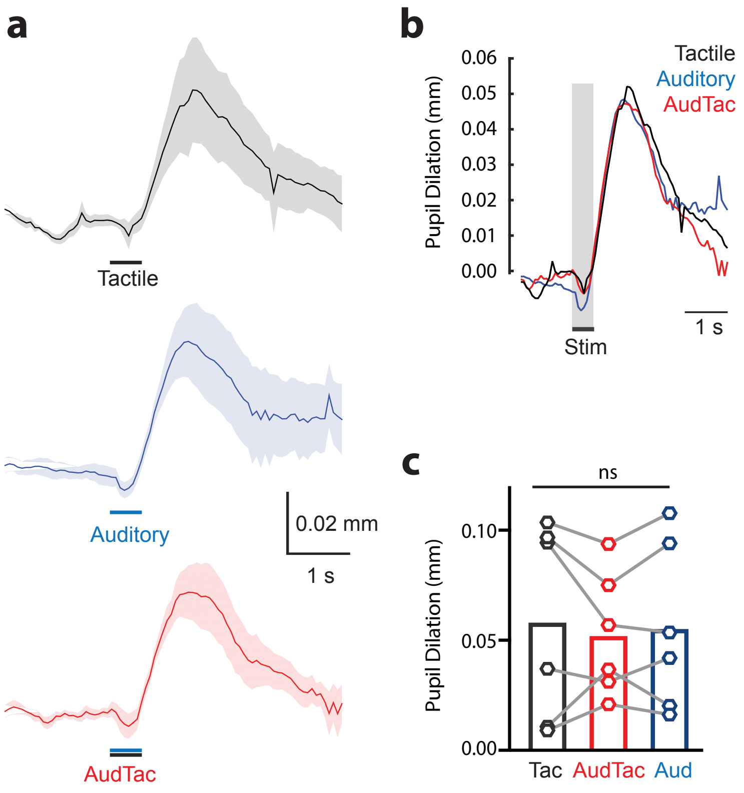

Supplementary Figure 5. Pupil tracking during tactile and auditory stimulus in naïve mice. a, Pupil dilation was measured during tactile (black; 200 Hz, 500 ms), auditory (blue; 2 - 50 kHz, 60 dB, 500 ms) and paired tactile and auditory (red) stimulus. Mean ± s.e.m. **b,** Overlay of average evoked pupil dilation. n = 6 mice. Grey, stimulus delivery. **c,** There was no significant difference between the dilation of the pupil evoked during tactile (black, 0.059 ± 0.02 mm), paired auditory and tactile (red, 0.052 ± 0.01 mm) and auditory (blue, 0.056 ± 0.02 mm). T v AT, p = 0.69; T v A, p = 0.56; TA v A, p = 0.69, n = 6 mice. Two-tailed Wilcoxon matched-pairs signed rank test.

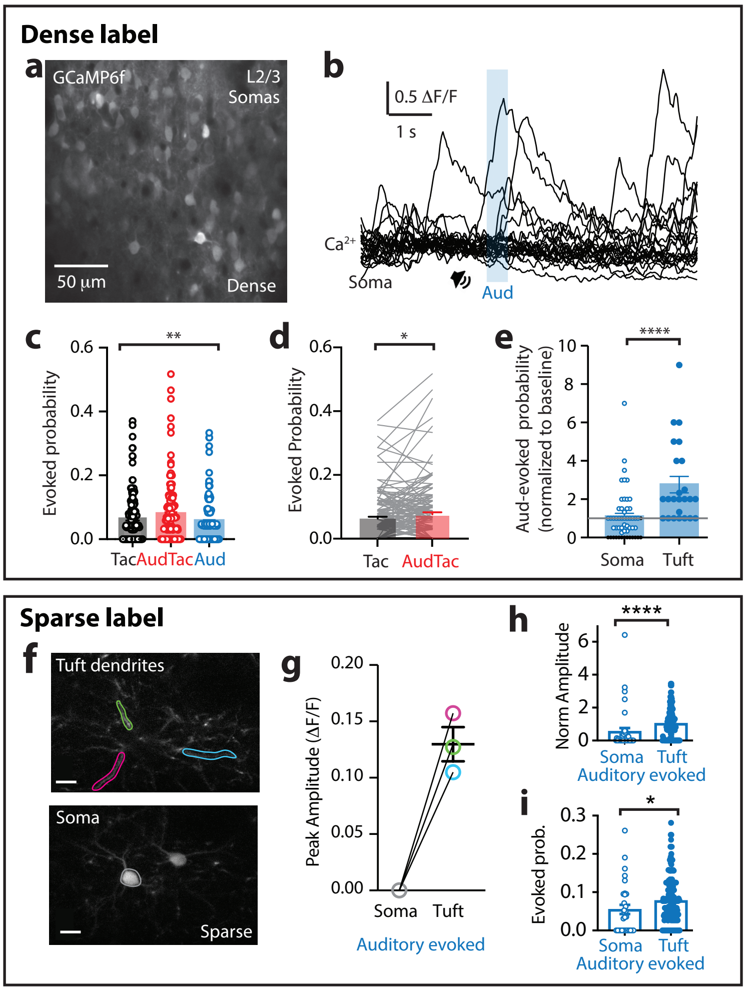

Supplementary Figure 6. Evoked activity in layer 2/3 pyramidal neuron somatas in the primary somatosensory cortex. a - e, In vivo two-photon Ca2+ imaging was performed in L2/3 pyramidal neurons densely labelled with GCaMP6f in naive mice. **a,** Example field of view. Two-photon image of dense labelling of somas located in layer 2/3. **b,** Overlay of Ca2+ responses during broadband auditory stimulation (2 - 50 kHz broadband, 70 dB) in an example neuron. Individual trials are overlaid. **c,** Probability of an evoked Ca2+ response in response to tactile (Tac, black), paired auditory and tactile (AudTac, red) and auditory (Aud, blue) stimuli ($p = 0.0099$; n = 181 somas, Friedman test). **d,** Direct comparison between the probably of an evoked Ca^{2+} response during tactile (Tac) and paired auditory and tactile (AudTac) stimulus ($p = 0.03$; Wilcoxon matched-pairs signed rank text). **e,** Comparison of the auditory-evoked Ca^{2+} response probability in soma (n = 53) and tuft dendrites (n = 24) from the same population of layer 2/3 pyramidal neurons (ie FOV) which were active during auditory input. Data is normalized to average baseline activity. $p < 0.0001$; Mann Whitney test. **f - i,** In vivo two-photon Ca2+ imaging was performed in L2/3 pyramidal neurons sparsely labelled with GCaMP6f in naive mice. **f,** Two-photon image of three tuft dendritic branches (top) and corresponding soma (bottom) from a single L2/3 pyramidal neuron. Here, tuft dendrites were traced to their soma of origin to compare auditory responses in the two compartments. Scale, 10 µm. **g,** Peak amplitudes of Ca^{2+} responses to auditory stimuli in the tuft dendrites (n = 3) and soma of the single neuron illustrated in (**f**). Colors correspond to the colored ROIs in (**f**). **h,** Normalised peak amplitudes of auditory-evoked responses from soma and dendrites from the same population of sparsely labelled L2/3 pyramidal neurons (tuft, 1.04 ± 0.09 vs soma, 0.55 ± 0.2, $p < 0.0001$, Mann Whitney test; n = 116 tuft dendrites, 38 soma; 6 mice). **i,** Probability of auditory stimuli evoking a Ca^{2+} response in soma and dendrites from the same population of sparsely labelled L2/3 pyramidal neurons (tuft, 0.78 ± 0.004; n = 197 vs soma, 0.55 ± 0.01, n = 31; $p = 0.016$, Mann Whitney test).* $p < 0.05$; ** $p < 0.01$; *** $p < 0.001$;****, $p < 0.0001$. All statistical tests are two-sided. Error bars represent S.E.M.

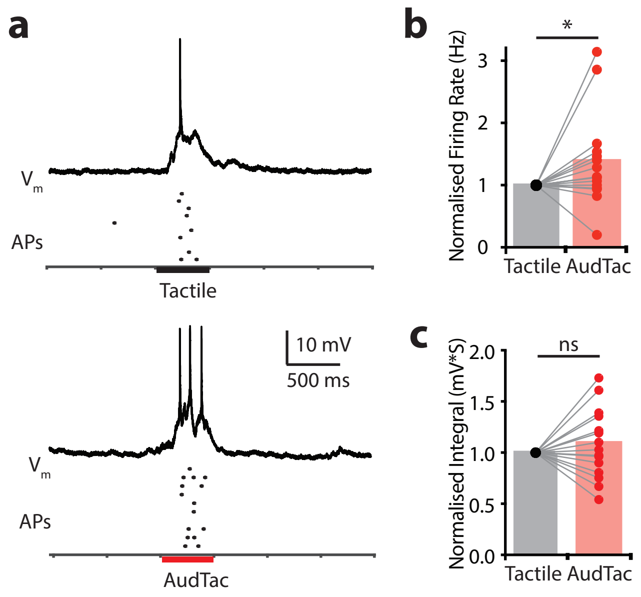

Supplementary Figure 7. Voltage recordings from L2/3 pyramidal neurons in forepaw S1 of anesthetized mice. Patch clamp electrophysiological recordings were performed from L2/3 pyramidal neurons in the primary somatosensory cortex of urethane anaesthetized mice. The contralateral forepaw was adhered to the vibrating button stimuli and the same sensory stimulation paradigm as the association task used in the dendritic recordings was presented to the mouse. The forepaw stimulation (200 Hz; 500 ms) was either presented alone or paired with auditory stimulation (broadband noise, 2 – 50 kHz, 60 dB, 500 ms). **a,** Example voltage trace and raster plot of action potentials (APs) from a layer 2/3 pyramidal neuron during forepaw tactile stimulation alone (Top; black line) and tactile forepaw stimulation paired with auditory stimulation (Bottom; Tactile + Auditory, red line). **b,** When paired with auditory stimulation, the number of action potentials evoked during tactile stimulation significantly increased by on average 37 ± 19 % (p = 0.049; n = 15 neurons, 12 mice). c, Paired auditory stimulation did not alter the tactile-evoked subthreshold voltage response (n = 15 neurons , 12 mice; p = 0.525). Statistical analysis; two-tailed Wilcoxon matched-pairs signed rank test. Error bars represent S.E.M.

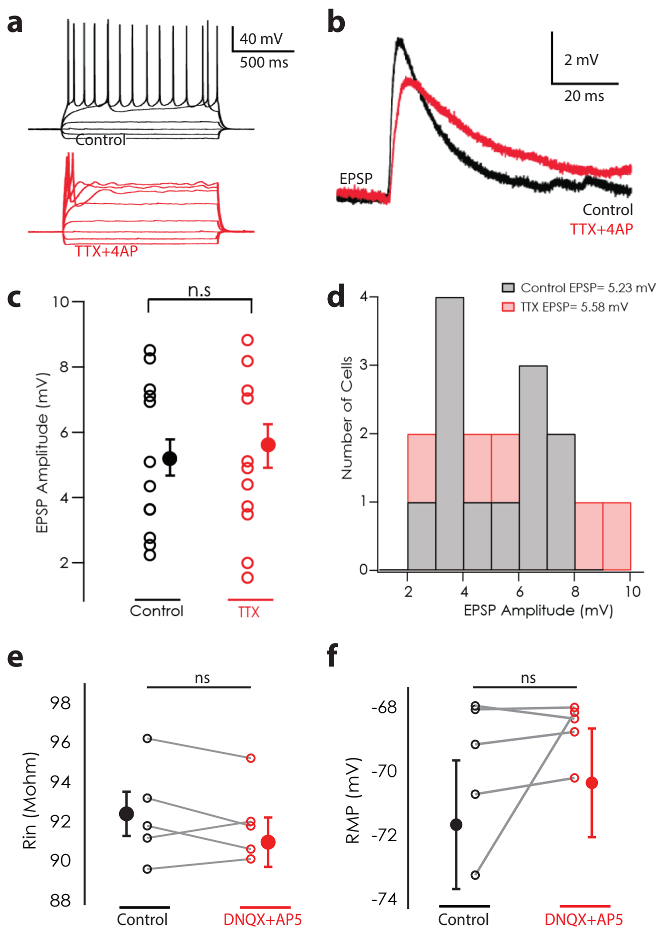

Supplementary Figure 8. Quantification of monosynaptic (TTX+4-AP) and synaptic (DNQX+AP5) responses. The photoactivatable opsin, ChR2, was injected into the auditory cortex (~100 nl; 200-500 µm below pia). After 10-14 days, patch clamp recordings were performed in layer 2/3 pyramidal neurons from brain slices of the primary somatosensory cortex. **a,** To test whether the layer 2/3 evoked potentials recorded in in the primary somatosensory cortex in response to photo-activation of auditory axons (ChR2, 470 nm) was monosynaptic, the antagonists of sodium (TTX) and potassium (4-AP) channels were bath applied. TTX+4AP (red) prevents action potential generation as illustrated during the current injection step protocol (50 pA, 1200 ms current steps). **b,** Example ChR2-evoked potentials in a layer 2/3 pyramidal neuron in the somatosensory cortex before (black, control) and after (red) bath application of TTX+4AP. **c,** Bath application of TTX+4-AP (red) did not significantly alter the amplitude of the photo-evoked (470 nm, 2 ms) potentials compared with control (black; $p = 0.69$; $n = 11$ cells; Two-tailed Mann-Whitney test). **d,** Histogram showing the average EPSP amplitudes for neurons during control (black) and TTX+4-AP (red). Bath application of the AMPA and NMDA channel blockers (DNQX and AP5 respectively; red) did not alter (**e**) input resistance (Rin; $p = 0.625$; $n = 5$ cells;) nor (**f**) resting membrane potential (RMP; $p = 0.722$; $n = 5$ cells). Two-tailed Wilcoxon matched-pairs signed rank test. Error bars represent S.E.M.

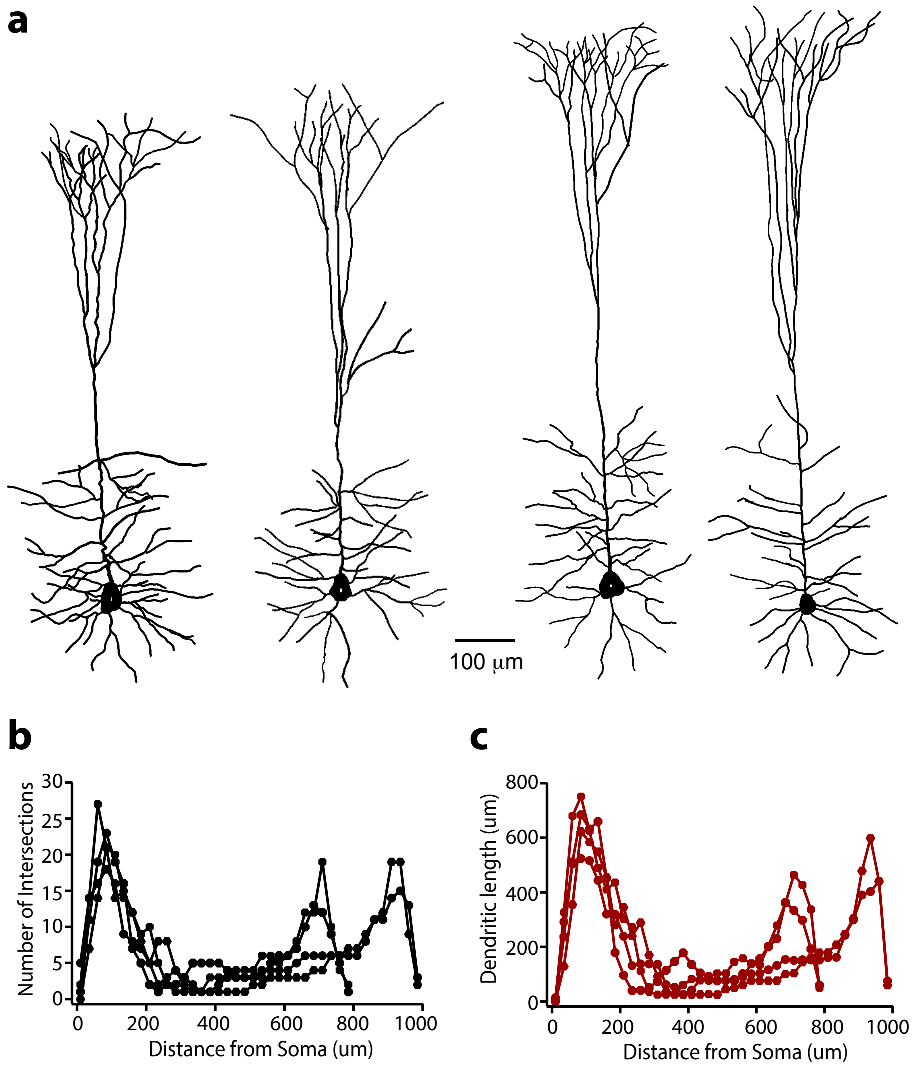

Supplementary Figure 9. Complete morphology of in vitro layer 5 pyramidal neurons. a. Layer 5 pyramidal neurons were filled with biocytin to visualise the dendritic morphology of the recorded neurons. All L5 pyramidal neurons recorded in vitro had intact dendritic arbors. There was no difference between **(b)** the number of intersections and **(c)** dendritic length. n = 4 neurons, 4 mice.

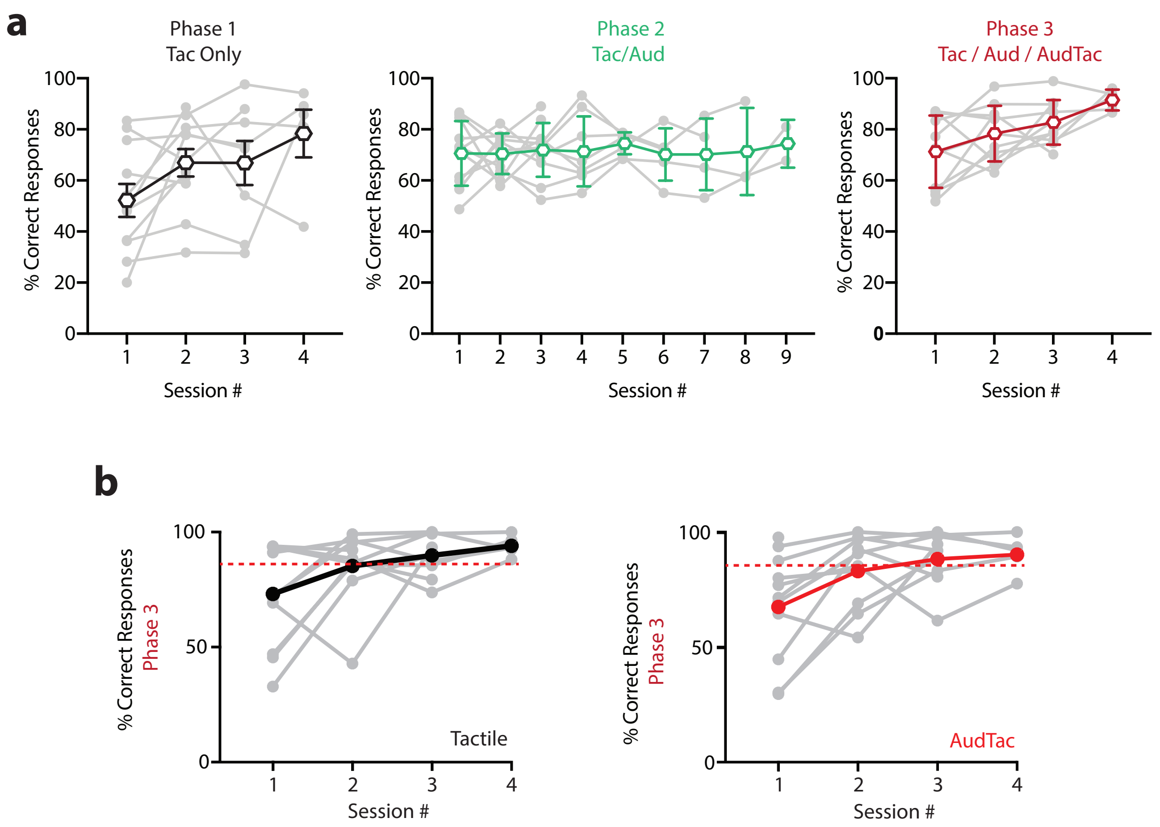

Supplementary Figure 10. Behavioral training paradigm and behavioral response characteristics. a, Overlay of correct performance during the training paradigm. Mice were trained in phases and only progress to next training phase once criteria (80% correct) has been obtained. 1) Tactile only (grey): mice are trained to associate forepaw tactile stimulation (200 Hz, 500 ms) with water. 2) Tac / Aud (green): mice are trained to withhold licking to auditory stimulus alone (broadband noise, 2 – 50 kHz, 60 dB, 500 ms). 3) Tac / Aud / AudTac (maroon): Paired tactile and auditory stimuli are presented to the mouse. Data points are average values for an entire session for a single mouse. Color line, average for all mice in the training phase. Incorrect performance was given time-outs in 100 ms intervals. **b,** There was no difference between the percentage of correct responses during learning duringTactile-trials (left) and AudTac-trials (right) in phase 3 of the training paradigm. Error bars represent S.E.M.

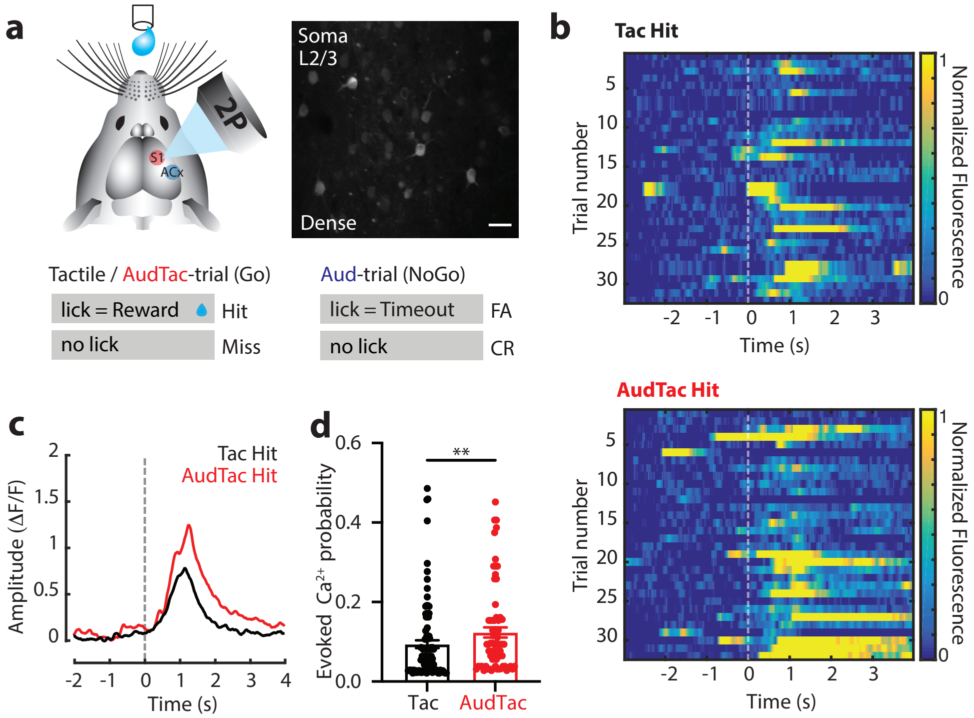

Supplementary Fig. 11. Somatic activity during the tactile-based goal-directed task. a, Example field of view. In vivo two-photon Ca^{2+} imaging of L2/3 pyramidal neuron somata was performed during expert performance in the tactile-based goal-directed task. Mice received a water reward if they licked in response to tactile stimulus alone (Tactile-trial; 200 Hz, 500 ms) and paired tactile and auditory stimulus (AudTac-trial). On random trials, mice were also presented with auditory stimulus alone (Auditory-trial; NoGo, 2 – 50 kHz; 500 ms) which was not rewarded (Correct Rejection, CR) and a time out was given if mice licked (False Alarm, FA). Scale bar = 20 μm **b,** Representative heatmaps of Ca^{2+} activity in a L2/3 pyramidal neuron soma during correct HIT performance in Tactile- (top) and AudTac- (bottom) trials. Fluorescence responses are normalized to maximum Ca^{2+} response. Stimulus was delivered at time 0 (dahsed line). **c,** Average Ca^{2+} response during Tactile- (black) and AudTac- (red) trials in a representative soma shown in (b). **d,** Evoked rate of somatic Ca^{2+} transients during Tactile- (black) and AudTac (red) trials (n = 98 / 71 somas; $p = 0.004$; Two-tailed Mann Whitney test). Error bars represent S.E.M.

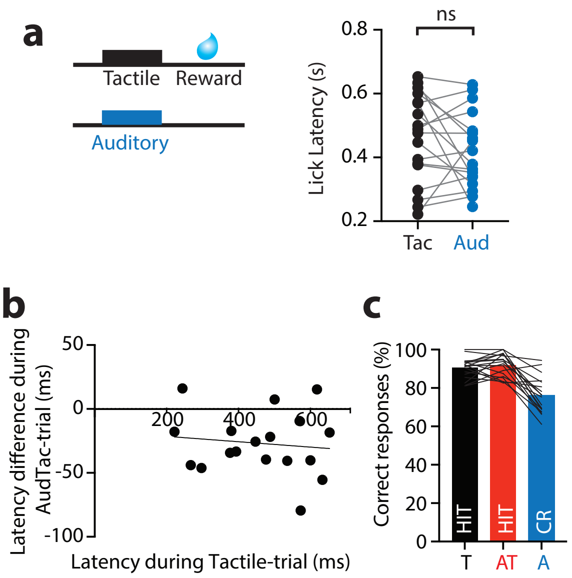

Supplementary Figure 12. Behavior controls. a. Left, Experimental paradigm. Right, There was no significant difference between the lick latency in response to tactile stimulus alone (black) and auditory stimulus alone ($p = 0.580$; n = 18 mice; two-tailed Wilcoxon matched-pairs signed rank test) in mice which had < 30 % false alarm rate. **b.** Correlation between the lick latency in tactile-trials and the influence of adding auditory input (Linear Regression, $R^2 = 0.014$; n = 18 mice). **c.** Percentage of correct responses in the tactile goal-directed task in mice (n = 18 mice).

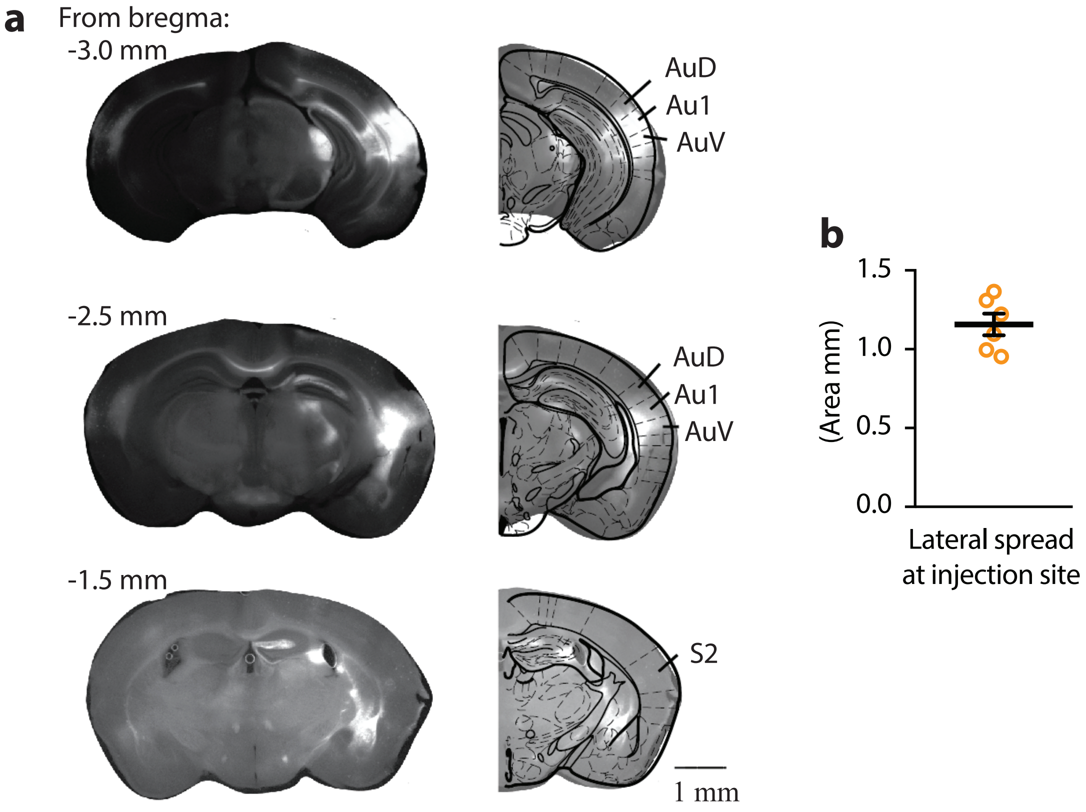

Supplementary Figure 13. Characterization of the viral spread for the ArchT cohort. Mice were injected with the inhibitory opsin Archaerhodopsin (AAV1.CAG.ArchT.GFP.WPRE.SV40) in the auditory cortex. **a.** Left, Flourescent images from an example mouse illustrating lateral spread of virus at -3.0, -2.5 and -1.5 mm from bregma. The injection site was at -2.50 mm from bregma. Right, Overlay of fluorescence and brain region categorization (from the Paxinos atlas). The virus did not spread towards S2 or other regions outside of the auditory cortex on the rostro-caudal axis. **b.** Lateral spread at the injection site for each mouse of the ArchT cohort (n = 6 mice). The local spread of the virus was contained within the auditory cortex and similar between each mouse. Error bars represent S.E.M.

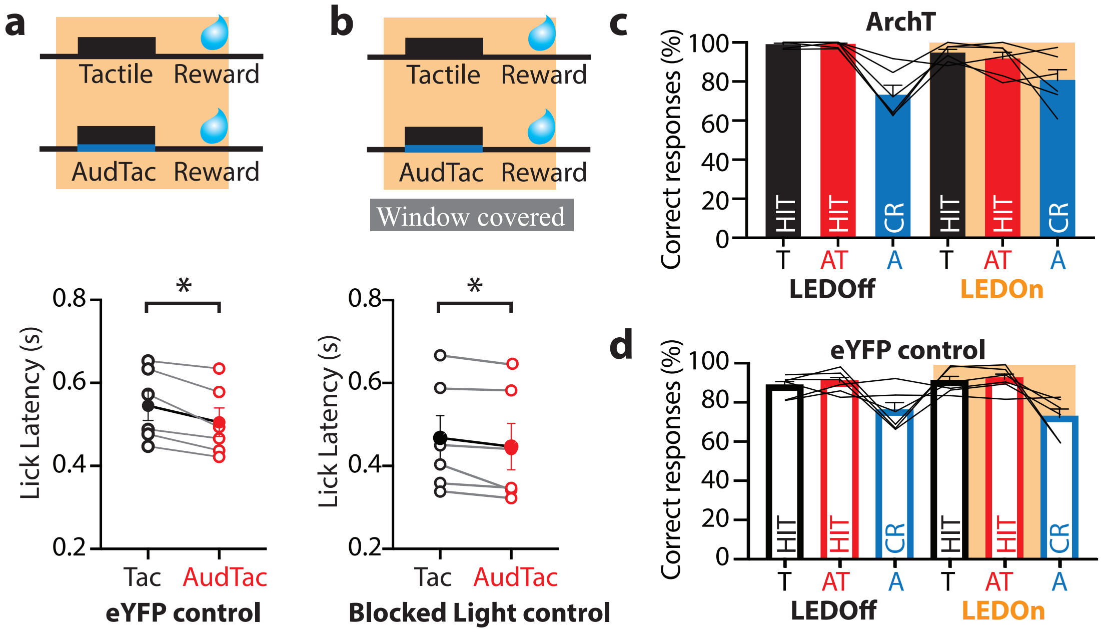

Supplementary Figure 14. Archaerhodopsin Controls. Mice were previously injected with the inhibitory opsin, Archaerhodopsin, in the auditory cortex. Axonal projections in the forepaw S1 were photoinhibited using a LED (590 nm). **a,** Top, Experimental paradigm. To test the influence of the LED protocol on behaviour, a control mus-eYFP was injected into the auditory cortex and the influence of LED was tested as the mouse performed the tactile goal-directed behavior. LED exposure in the sham mouse did not influence the enhanced response latency ($p = 0.0312$; $p_{shuffled} = 0.563$; $n = 6$ mice; 35 trials$_{av}$; Two-tailed Wilcoxon matched-pairs signed rank test). **b,** Top, Experimental paradigm. To test the influence of the LED protocol on behaviour, the chronic window was covered with inert opaque silicone during the LED behavioural paradigm. Bottom, During the blocked LED protocol, the lick latency was significantly decreased, illustrating LED itself did not influence behavior ($p = 0.03$; $p_{shuffled} = 0.438$; $n = 6$ mice; 35 trials$_{av}$; Two-tailed Wilcoxon matched-pairs signed rank test). **c.** Percentage of correct responses in the tactile goal-directed task in mice previously injected with Archaerhodopsin during LED Off and LED On ($n = 6$ mice). **d,** Percentage of correct responses in the tactile goal-directed task in mice previously injected with control fluorophore mus-eYFP during LED Off and LED On ($n = 6$ mice). Error bars represent S.E.M.
