# Supplementary material for: Auditory input enhances somatosensory encoding and tactile goal-directed behavior
Source: Nat Commun. 2021 Jul 23;12:4509. doi: 10.1038/s41467-021-24754-w (PMC8302566; doi:10.1038/s41467-021-24754-w)
Supplement: Supplementary file 3 — Reporting summary [file 41467_2021_24754_MOESM3_ESM.pdf]

nature portfolio

Corresponding author(s): Lucy Palmer

Last updated by author(s): Jun 22, 2021

Reporting Summary

Nature Portfolio wishes to improve the reproducibility of the work that we publish. This form provides structure for consistency and transparency in reporting. For further information on Nature Portfolio policies, see our Editorial Policies and the Editorial Policy Checklist.

Statistics

For all statistical analyses, confirm that the following items are present in the figure legend, table legend, main text, or Methods section.

n/a	Confirmed	
☐	☒	The exact sample size (n) for each experimental group/condition, given as a discrete number and unit of measurement
☐	☒	A statement on whether measurements were taken from distinct samples or whether the same sample was measured repeatedly
☐	☒	The statistical test(s) used AND whether they are one- or two-sided<br>*Only common tests should be described solely by name; describe more complex techniques in the Methods section.*
☐	☒	A description of all covariates tested
☐	☒	A description of any assumptions or corrections, such as tests of normality and adjustment for multiple comparisons
☐	☒	A full description of the statistical parameters including central tendency (e.g. means) or other basic estimates (e.g. regression coefficient) AND variation (e.g. standard deviation) or associated estimates of uncertainty (e.g. confidence intervals)
☐	☒	For null hypothesis testing, the test statistic (e.g. F, t, r) with confidence intervals, effect sizes, degrees of freedom and P value noted<br>*Give P values as exact values whenever suitable.*
☒	☐	For Bayesian analysis, information on the choice of priors and Markov chain Monte Carlo settings
☒	☐	For hierarchical and complex designs, identification of the appropriate level for tests and full reporting of outcomes
☒	☐	Estimates of effect sizes (e.g. Cohen's d, Pearson's r), indicating how they were calculated

Our web collection on statistics for biologists contains articles on many of the points above.

Software and code

Policy information about availability of computer code

Data collection	ScanImage software (Vidrio Technologies) was used to record Ca2+ transients. Custom-written Igor Pro (Wavemetrics) software was used for the acquisition of whole-cell recordings. Behaviour was recorded using Bpod (Sanworks).
Data analysis	Ca2+ imaging data were initially visualized in ImageJ (FIJI). Custom-written MATLAB (MathWorks) software was used for analysis of Ca2+ imaging data. Custom-written Igor Pro (Wavemetrics 6.2) software was used for the analysis of whole-cell recordings. Behaviour was analyzed using Bpod software (Gen 2; Sanworks).

For manuscripts utilizing custom algorithms or software that are central to the research but not yet described in published literature, software must be made available to editors and reviewers. We strongly encourage code deposition in a community repository (e.g. GitHub). See the Nature Portfolio guidelines for submitting code & software for further information.

Data

Policy information about availability of data

All manuscripts must include a data availability statement. This statement should provide the following information, where applicable:

- Accession codes, unique identifiers, or web links for publicly available datasets
- A description of any restrictions on data availability
- For clinical datasets or third party data, please ensure that the statement adheres to our policy

The raw source data and analysis code contributing to this study are available from the corresponding author on request.

Field-specific reporting

Please select the one below that is the best fit for your research. If you are not sure, read the appropriate sections before making your selection.

- [x] Life sciences
- [ ] Behavioural & social sciences
- [ ] Ecological, evolutionary & environmental sciences

For a reference copy of the document with all sections, see nature.com/documents/nr-reporting-summary-flat.pdf

Life sciences study design

All studies must disclose on these points even when the disclosure is negative.

Sample size	No pre-determined sample sizes were calculated prior to experiments. Statistical significance tests were performed on experimental results according to type (paired/non-paired) and distribution.
Data exclusions	No data was excluded from this study.
Replication	Behavioral experiments were performed by different authors, illustrating the robust nature of the findings.
Randomization	Stimuli was randomly presented to the mice.
Blinding	Investigators were blinded during the analysis procedures. Analysis procedures were either fully or semi-automated (ie behavioral outcome and fluorescence/voltage detection), thus eliminating analysis bias.

Reporting for specific materials, systems and methods

We require information from authors about some types of materials, experimental systems and methods used in many studies. Here, indicate whether each material, system or method listed is relevant to your study. If you are not sure if a list item applies to your research, read the appropriate section before selecting a response.

Materials & experimental systems

n/a	Involved in the study	
x		Antibodies
x		Eukaryotic cell lines
x		Palaeontology and archaeology
	x	Animals and other organisms
x		Human research participants
x		Clinical data
x		Dual use research of concern

Methods

n/a	Involved in the study	
x		ChIP-seq
x		Flow cytometry
x		MRI-based neuroimaging

Animals and other organisms

Policy information about studies involving animals; ARRIVE guidelines recommended for reporting animal research

Laboratory animals	C57BL/6 mice (P25-63) of either sex were used in this study
Wild animals	This study did not involve wild animals
Field-collected samples	This study did not involve samples collected from the field
Ethics oversight	All experiments were conducted in strict accordance with the Code of Practice for the Care and Use of Animals for Scientific Purposes (National Health and Medical Research Council, Australia). The guidelines were given by the veterinary office and approved by the Animal Ethics Committees of The Florey Institute of Neuroscience and Mental Health, University of Melbourne, and of the Australian National University.

Note that full information on the approval of the study protocol must also be provided in the manuscript.
